# Immune Reconstitution Following Autologous Hematopoietic Stem Cell Transplantation for Multiple Sclerosis: A Review on Behalf of the EBMT Autoimmune Diseases Working Party

**DOI:** 10.3389/fimmu.2021.813957

**Published:** 2022-02-01

**Authors:** Maria Teresa Cencioni, Angela Genchi, Gavin Brittain, Thushan I. de Silva, Basil Sharrack, John Andrew Snowden, Tobias Alexander, Raffaella Greco, Paolo A. Muraro

**Affiliations:** ^1^ Division of Neurology, Department of Brain Sciences, Imperial College London, London, United Kingdom; ^2^ Department of Neurology, Neurology Unit, Istituto di Ricovero e Cura a Carattere Scientifico (IRCCS) San Raffaele Scientific Institute, Vita-Salute San Raffaele University, Milan, Italy; ^3^ South Yorkshire Regional Department of Infection and Tropical Medicine, Sheffield Teaching Hospitals National Health Service (NHS) Foundation Trust, Sheffield, United Kingdom; ^4^ Institute for Translational Neuroscience and Sheffield Neuroscience Biomedical Research Centre (BRC), Sheffield, United Kingdom; ^5^ Department of Infection, Immunity and Cardiovascular Disease, The University of Sheffield, Sheffield, United Kingdom; ^6^ Department of Haematology, Sheffield Teaching Hospitals National Health Service (NHS) Foundation Trust, Sheffield, United Kingdom; ^7^ Department of Oncology and Metabolism, The University of Sheffield, Sheffield, United Kingdom; ^8^ Charité – Universitätsmedizin Berlin, Corporate member of Freie Universität Berlin and Humboldt-Universität zu Berlin, Department of Rheumatology and Clinical Immunology, Berlin, Germany; ^9^ Deutsches Rheuma-Forschungszentrum, ein Leibniz Institut, Berlin, Germany; ^10^ Unit of Haematology and Bone Marrow Transplantation, Istituto di Ricovero e Cura a Carattere Scientifico (IRCCS) San Raffaele Scientific Institute, Vita-Salute San Raffaele University, Milan, Italy

**Keywords:** hematopoietic stem cell (HSC) transplantation, disease-modifying therapies (DMT), immune reconstitution, immunological memory, vaccination

## Abstract

Multiple sclerosis (MS) is a central nervous system (CNS) disorder, which is mediated by an abnormal immune response coordinated by T and B cells resulting in areas of inflammation, demyelination, and axonal loss. Disease-modifying treatments (DMTs) are available to dampen the inflammatory aggression but are ineffective in many patients. Autologous hematopoietic stem cell transplantation (HSCT) has been used as treatment in patients with a highly active disease, achieving a long-term clinical remission in most. The rationale of the intervention is to eradicate inflammatory autoreactive cells with lympho-ablative regimens and restore immune tolerance. Immunological studies have demonstrated that autologous HSCT induces a renewal of TCR repertoires, resurgence of immune regulatory cells, and depletion of proinflammatory T cell subsets, suggesting a “resetting” of immunological memory. Although our understanding of the clinical and immunological effects of autologous HSCT has progressed, further work is required to characterize the mechanisms that underlie treatment efficacy. Considering that memory B cells are disease-promoting and stem-like T cells are multipotent progenitors involved in self-regeneration of central and effector memory cells, investigating the reconstitution of B cell compartment and stem and effector subsets of immunological memory following autologous HSCT could elucidate those mechanisms. Since all subjects need to be optimally protected from vaccine-preventable diseases (including COVID-19), there is a need to ensure that vaccination in subjects undergoing HSCT is effective and safe. Additionally, the study of vaccination in HSCT-treated subjects as a means of evaluating immune responses could further distinguish broad immunosuppression from immune resetting.

## Introduction

Multiple sclerosis (MS) is a chronic demyelinating disease of the central nervous system (CNS) characterized by a dysregulation of self-tolerance toward myelin and persistent activation of autoreactive cells (1–3). Infiltration of peripheral self-reactive cells in the CNS and bystander immune activation of microglia and resident macrophages damages the myelin sheaths causing demyelination, axonal transection, chronic inflammation, and over time progressive neuronal damage. Infiltrating cells show a memory and effector memory phenotype with pro-inflammatory and cytotoxic features (4). Effective treatment strategies in MS are mostly based on lympho-depletion or blockade of immune cell recirculation, but the aim of disease remission is not met in every patient; and long-term drug administration results in exposure to risks including progressive multifocal leukoencephalopathy (PML), hypogammaglobulinemia, and autoimmune thrombocytopenia as well as other secondary autoimmune diseases (5). Autologous hematopoietic stem cell transplantation (HSCT) has demonstrated long remission and neurological improvement with acceptable safety for selected patients with highly active, relapsing–remitting MS (RRMS), in which it is currently considered a standard treatment according to EBMT guidelines (6, 7). The rationale of HSCT is to remove pathogenic cells with a myelo- or lymphoablative conditioning regimen and allow immune reconstitution from myeloid or lymphoid progenitor cells. Thymus-dependent T cell regeneration and immune regulation mediated by T and natural killer (NK) cells constitute the major identified pathways influencing immune reconstitution after HSCT for treatment of autoimmune diseases. The mechanism underlying the long clinical remission post-HSCT remains unknown. Immune memory persistence could be the cause of treatment failure in MS especially in patients with high degree of inflammation. Recent discoveries have pointed out that memory stem cells (Tscm) with a hybrid phenotype of stem cells and effector memory cells contribute to chronic inflammation and tissue damage in autoimmune disorders. In this article, we highlight current knowledge and provide a perspective on studies with potential to advance our understanding of the mechanisms of HSCT as therapy for people with MS. We also discuss how protective immunity following vaccination before and after autologous HSCT can serve as a surrogate marker of immunological memory.

## Disease-Modifying Treatments in MS

Disease-modifying therapies (DMTs) for MS have significantly expanded in the last 30 years, posing new challenges for clinicians and patients. The majority of DMTs are used in RRMS, and among second-line and immune-suppressive therapies, we can distinguish between immune-sequestering (natalizumab) and lympho-depleting treatments (rituximab/ocrelizumab/ofatumumab, alemtuzumab, and cladribine).

Natalizumab (NAT) is a monoclonal antibody which binds the VLA-4 integrin α4 chain (CD49d), thereby inhibiting leukocyte migration into the CNS and gut parenchyma. NAT efficacy for RRMS has been demonstrated by randomized clinical trials (RCTs) (8, 9) and real-world data (10, 11). However, NAT therapeutic success has been hampered by the risk of progressive multifocal leukoencephalopathy (PML), a diffuse demyelinating disease caused by polyomavirus reactivation referred to as John Cunningham virus (JCV) (12). The risk of developing PML ranges from 0.09 to 11.1 cases per 1,000 per year (12). While it is reduced with NAT-extended interval dosing (13), concerns persist in undertaking this treatment in JCV-positive patients. Following treatment discontinuation (often due to PML risk), disease reactivation/rebound is observed in up to 27.9% of patients within 6 months and disability accumulation in 37% of cases (14).

Rituximab (RTX), a chimeric mouse–human monoclonal antibody, and ocrelizumab (OCR) and ofatumumab (OFA), second-generation humanized monoclonal antibodies, are anti-CD20-depleting therapies. Both induce the depletion of a broad range of B cells, sparing plasmablasts and plasma cells, suggesting an antibody-independent mechanism of action. Despite several randomized clinical trials (RCTs) and real-world data demonstrating RTX efficacy (15), only OCR and OFA have been approved for RRMS based on the results of the OPERA I and II studies (16, 17). Compared to IFNβ-1a, OCR and OFA are associated with a modestly higher risk of infections, mainly respiratory infections, varicella-zoster, and herpes simplex. Moreover, anti-CD20 monoclonal antibodies pose a high risk of HBV-associated hepatitis, liver failure (18, 19), and an increased risk of severity of SARS-CoV-2 infection (20). Despite B cell-depleting therapies being administered as pulsed infusions every 1–6 months, the related B-cell immunosuppression must be considered chronic, with consequences in terms of cumulative adverse events. Therefore, long-term data are required to explore any potential increased rate of malignancies, chronic hypogammaglobulinemia, or infection.

Alemtuzumab (ALEM), a monoclonal antibody against the CD52 surface antigen, induces rapid lymphocyte depletion. The CARE-MS I and II trials demonstrated ALEM efficacy compared with IFNβ-1a in treatment-naïve RRMS patients and those who had failed first-line therapies (21, 22). The extension studies, with a 5-year follow-up (23, 24), have shown that 68.5% and 59.8% of patients required only the two initial courses of ALEM to maintain treatment efficacy, and >95% of patients received no other DMTs. A high infection risk has been reported (67.3% in CARE-MS I and 76.8% in CARE-MS II), as well as the development of secondary autoimmune disorders which may be related to the rapid B cell recovery in absence of T cell immune regulation (25).

Cladribine (CdA), an oral DMT, is a purine analogue that, when metabolized to its active form, is concentrated in lymphocytes and monocytes while sparing other cells. The phase III CLARITY study showed high efficacy in the treatment of RRMS compared with placebo (26). In the extension study, no additional benefit was observed with four consecutive annual courses of treatments compared to two (26). Nevertheless, post-marketing studies have downsized CdA effectiveness compared to the highly efficacious therapies (27). Severe side effects including risk of myelosuppression, opportunistic infections, nephrotoxicity, and possible increased risk of malignancy were reported (5).

## Autologous HSCT as Treatment for MS

### Highlights on Outcomes After HSCT in Progressive and Active Relapsing MS

The clinical experience of HSCT in MS started in 1995 and involved European and North American centers (28). Initially, patients considered suitable for the trials showed high disability, advanced progressing disease, unresponsiveness to conventional treatments, and, in some, disease activity in the year preceding the enrolment, evaluated by clinical deterioration or/and evidence of gadolinium (Gd)-enhanced disease lesions in magnetic resonance imaging (MRI). Results from these studies reported a remarkable reduction or complete abolition of disease activity established by a decreased number of Gd-positive lesions starting immediately after mobilization with cyclophosphamide (Cy) and further declining in the months after conditioning therapy (28–34). The effectiveness of HSCT in progressive MS was estimated by measuring the evolution of disability measured by the Expanded Disability Status Scale (EDSS) and showed a failure of 40% and 52% at 3 and 6 years, respectively, with a deterioration of neurological function (32, 35), while disease progression-free survival at 15 years was 44% with active CNS disease pre-transplant and 10% for those without (36). A retrospective analysis of long-term (median follow-up of 6.6 years) HSCT outcomes in 281 patients (78% of them with progressive MS) clearly showed that younger age, relapsing form of MS, fewer prior immunotherapies, and lower baseline EDSS scores were factors associated with better outcomes. Progressive MS (PMS) was associated with neurological progression after transplant compared to relapsing forms of disease (HR, 2.33; 95% CI, 1.27–4.28) (37). In a prospective phase II clinical trial of HSCT for treatment refractory MS including patients with RRMS (57%) and with secondary progressive MS (SPMS) (43%), the event-free survival (EFS), defined as freedom from MS relapses, was 60% (70% for RRMS), without evidence of relapse, disability progression, or new MRI lesions, after a median follow-up of 36 months (38). Conclusions can be made that patients with RRMS respond more favorably to HSCT than SPMS even when patients with SPMS have an “active MRI.”

In some studies, HSCT was used to treat patients with a diagnosis of “malignant” RRMS, characterized by short duration of disease and recurrent and severe relapses (39–45) with a clear suppression or stabilization of the disease course. Subsequent studies increasingly or exclusively treated with HSCT patients with relapsing–remitting MS (RRMS). One single-center study reported that HSCT in subjects in the early course of RRMS, which failed at least 6 months of interferon-β; induced a significant EDSS improvement, at 6 and 12 months and 2 and 4 years, 100% of progression-free survival and 76% of relapse-free survival (45). Those results indicated that HSCT in patients with active disease removes inflammation and generates a long-term remission, improving neurological condition, potentially stopping or delaying neurodegenerative processes. In a phase 2 trial termed autologous HSCT with high-dose immunosuppressive therapy (HDIT) performed in RRMS (HALT-MS), EFS, absence of new MRI lesions, and neurological worsening in treated participants approached 70% after a median 62-month follow-up (41). The individual components of the composite outcome showed 91.3% of EDSS progression-free survival, 86.9% of clinical relapse-free survival, and 86.3% of MRI event-free survival (41).

Studies with long-term follow-up demonstrated a durable remission, and stabilization of a clinical course is sustained long beyond the immunosuppressive effect of chemotherapy, confirming a better outcome in patients with relapsing forms of MS (37, 46).

Despite the efficacy of HSCT in MS, it remains associated with a treatment-related mortality risk ranging from none in some clinical trials and reports (41, 47, 48) to around 0.2% (49, 50), 1.4% (51), 2.5% (52) in larger series up to 4% in one trial of patients treated with a high intensity myeloablative regimen (46). Overall, there has been reduction in transplant-related mortality (TRM) over time (51, 53), probably related to better patient selection and choice of conditioning regimen, which has permitted acceptance within the neurological community. Even with successful treatment, short- and longer-term adverse events are recognized, including infection, herpes virus reactivation (Epstein–Barr virus, cytomegalovirus) (48), secondary autoimmunity (up to 10%), endocrinopathy, and late cancers (54, 55). However, in the last decade, the safety of the procedure has shown a marked improvement, thanks to a growing experience in selecting the most appropriate patients to transplant, and advances in conditioning and support regimens. According to recent EBMT (56) data, rates of TRM have been falling to around 1% or below, in recent years. The demographics and outcomes from studies of HSCT in MS have been recently reviewed (57).

### HSCT Versus DMT

There are only two available RCTs comparing HSCT to current DMTs in MS, both with methodological limitations. The ASTIMS study compared myeloablative HSCT to mitoxantrone (MTX) in patients with RRMS (33%) or PMS (67%) (58). Regardless of disease subtype, the HSCT group demonstrated a significant reduction in new T2 lesions at 4 years, complete suppression of new Gd+ lesions (versus 56% of MTX patients), and significantly reduced annualized relapse rate (0.19 vs. 0.6 for MTX). However, HSCT did not demonstrate EDSS stabilization or improvement: this might be related to the high proportion of progressive patients, known to respond less favorably (59). The MIST study was an open-label trial of 110 RRMS patients, randomized 1:1 to receive non-lymphoablative HSCT or an FDA-approved DMT, based on the treating neurologist’s judgment (47). At 5 years, disease progression was remarkably suppressed in the HSCT arm (9.71% versus 75.3%) with similar impressive reductions in relapses (15.4% versus 85.2%). No evidence of disease activity (NEDA), defined as no progression, no relapses, and no new or enlarging lesions on magnetic resonance imaging, was seen in 78.5% of HSCT patients versus 2.97% in the DMT arm. Patients in the DMT group who experienced progression despite 1 year of treatment were crossed over (n = 31) to HSCT, and significant comparable outcomes to the patients initially randomized to HSCT were seen in EDSS scores and mean T2-weighted lesion volume on MRI. The MIST study has several limitations (1): incomplete follow-up data in the DMT arm, due to treatment crossover, (2) ALEM was excluded from use in the DMT group because of drug-related persistent lymphopenia and autoimmune disorders. OCR, OFA, and CdA were not licensed at the time the study was opened to recruitment. Clinical outcomes from ASTIMS and MIST studies are summarized in **Table 1**. While waiting for a direct comparison between the efficacy of HSCT and that of approved highly effective DMTs, some information can be cautiously derived by considering the degree of NEDA achieved in clinical trials of HSCT compared with that of other approved DMTs. Comparisons between different RCTs must be made with caution considering different population characteristics and follow-up schedules. A cross-sectional analysis reveals that the proportion of patients for whom NEDA was achieved at 2 years was 7%–16% among those who received placebo, 13%–27% among patients who received IFNβ-1a, and 22%–48% among patients who received other active drugs (dimethyl fumarate, fingolimod, NAT, ALEM, OCR); among patients who underwent HSCT, the NEDA proportion was considerably higher, at 70%–92% (60). In a retrospective, single-center, real-world study comparing the efficacy and safety of HSCT vs. ALEM in aggressive RRMS patients, HSCT seems to be superior to ALEM in inducing complete disease control (NEDA 75% versus 56%; p = 0.023) and in promoting short-term disability improvement (61). Available evidence does not allow the identification of patients who would benefit from early aggressive therapy versus an escalation approach. Despite highly effective DMTs providing significant control of disease activity, they carry the risk of serious adverse events. Immune-suppressive DMTs are associated with an increased risk of mild to moderate infections and with reactivation of latent pathogens. The risk of herpesvirus and tuberculous-related diseases is increased with immunosuppressive therapies; similarly, hepatitis B virus (HBV) reactivation is a risk with anti-CD20 DMTs.

**Table 1:** Selected clinical outcomes from the two randomized controlled trials of HSCT vs. DMT.

	ASTIMS*^a^*		MIST study*^b^*	
	HSCT	Mitoxantrone	HSCT	DMT
Magnetic resonance imaging	New T2-weighted lesions^		Mean change in T2-weighted lesion volume^	
	0%	56%	-32%	+34%
Relapses	Annualized relapse rate^		New relapses^	
	0.19	0.6	15%	85%
Clinical progression	Increase in EDSS*		1-point increase in EDSS score^	
	57%	48%	29%	75%
Limitations	Inclusion of patients with PMS (67%)		Limited follow-up data in DMT arm, due to treatment crossover	
	Missed inclusion of currently used highly effective DMTs in the control arm			

^a^Mancardi GL, Sormani MP, Di Gioia M, Vuolo L, Gualandi F, Amato MP, et al. Autologous haematopoietic stem cell transplantation with an intermediate intensity conditioning regimen in multiple sclerosis: the Italian multi-centre experience. Mult Scler. 2012;18(6):835-42.9. ^b^Burt RK, Balabanov R, Burman J, Sharrack B, Snowden JA, Oliveira MC, et al. Effect of Nonmyeloablative Hematopoietic Stem Cell Transplantation vs. Continued Disease-Modifying Therapy on Disease Progression in Patients with Relapsing-Remitting Multiple Sclerosis: A Randomized Clinical Trial. Jama. 2019;321(2):165-74.

DMT, disease-modifying treatment; HSCT, Autologous hematopoietic stem cell transplantation; PMS, progressive MS; EDSS, Expanded Disability Status Scale.

Symbol * = no statistical difference, ^ = statistical difference.

### Studies of Immune Reconstitution Following Autologous HSCT in MS

Since 2000, detailed immunological studies have started to examine the immune effects and potential mechanisms of action of autologous HSCT for treatment of autoimmune disease. Focusing on contributions from studies in treated MS patients, we first review here some key results on studies of adaptive and innate immunity. We next identify some important questions and outline future studies that could address them.

### Adaptive Immunity

#### T Cells

In one of the early studies, Muraro et al. showed that CD3^+^ T cell levels were reported to have recovered at 6 months after high-intensity myelo- and immunoablative HSCT with different kinetics of immune reconstitution of CD4^+^ and CD8^+^ T cells (62); CD4^+^ T cells recovered gradually compared to CD8^+^ T cells, and the CD4/CD8 ratios decreased at 6 months and reached the baseline levels at 1 year (62). The longitudinal analysis of the frequency of T cell subpopulations after HSCT showed a phenotypic renewal of the T helper cell compartment. In the CD4+ compartment, memory (M) T cells (CD45RA^-^CD45RO^+^CD27^+^) predominate at the baseline and are replaced by naïve T cells (CD45RA^+^CD45RO^-^CD27^+^) at 2 years after therapy with a 76% reduction of T_M_/T_N_ ratios (62). The *de novo* generated CD4^+^ T cells in the peripheral blood (PB) display features of thymic origin, such as increased co-expression levels of CD31 or T-cell receptor excision circles (TREC). counts and a strong significant correlation of frequency of CD4-naïve T cells and CD4 RTE T cells at the 1- and 2-year follow-up. Analysis of a single T cell receptor (TCR) repertoire at the single clone level by sequencing of TCRβ transcripts of sorted PB CD4 T cells demonstrated increased repertoire diversity compared to the pre-HSCT (62). The dominant preexistent TCR clones were completely depleted after the conditioning regimens and replaced by clones with a new repertoire (63). Contrary to the CD4 compartment, the CD8+ pool showed an incomplete renewal of clonal specificities with the persistence of preexisting clones (62). The proportion of subpopulations did not change compared to the baseline. The only difference was on effector memory terminally differentiated CD8 T cells that expressed senescence phenotype CD28^-^CD57^+^CD95/FAS^+^CD45RA^+^CD45RO^+^CD27^-^ (62). High-throughput deep TCRβ chain sequencing on CD8 T cell clones before and after HSCT showed that the CD8 compartment post-HSCT was predominantly constituted by a selective expansion of dominant preexistent TCR clones (63).

Long-term TCR repertoire reconstitution was examined in matched CSF and PB CD4 and CD8 T cell clones before and up to 4 years after HSCT (64). The reconstituted repertoire in CSF included a majority of new T cell clonotypes generated from hematopoietic stem cells (HSC) and a smaller population of clones generated from memory T cells in PB preexisting before the therapy and resistant to immune ablation (64). The persistence of those clones in patients with a sustained remission of inflammatory disease activity led to the conclusion that they are not self-reactive pathogenic mediators or are not able to induce disease activity in the new conditions.

An immunophenotyping study conducted by CyTOF mass cytometry and performed on cryopreserved PBMCs from patients with MS treated with HDIT/HSCT (HALT-MS) showed a redistribution of T cell subsets. The analysis showed an increased proportion of effector memory (CD45RA-CCR7-) and late effector (CD45RA+CCR7-) subtypes associated with reduction of naïve and CM at 2 months and a return of subsets at baseline levels at 1 and 2 years post-HSCT (65). The immune reconstitution was compared in patients that had long remission of disease to those that had relapses to define biomarkers associated with disease activity. The 5-year positive outcome from HSCT was related to higher absolute cell counts of memory and effector memory CD4 and CD8 T cells in PB at the baseline, and it was suggested as a biomarker (65). These results support that an immune resetting of the memory phenotype in the T cell compartment is relevant for the resolution of inflammation.

The immune reconstitution of T cells after non-myeloablative HSCT reported a decrement of total lymphocyte count up to the first year after treatment (66). CD4 T cells within the total T cell population remained reduced for the entire 2-year follow-up whereas non-significant differences were detected in the CD8 T cell pool (66). Significant changes in immunophenotyping were observed only in the CD8 compartment. Expansion of memory cells was reported at 6 months and 1–2 years posttreatment with decrement of naïve cells at the same time points (66).

#### Myelin Antigen-Specific T Cells

CD4 and CD8 T cell response (proliferation and cytokine production) to multiple myelin epitopes including whole myelin basic protein (MBP), myelin oligodendrocyte glycoprotein (MOG), and peptide pools derived from MBP and myelin proteolipid protein (PLP) remerged in the PB after high-intensity HSCT despite the ablation of T cell response to the memory antigen tetanus toxoid (TT) (67). Furthermore, the reconstituted MBP-reactive T cells 12 months post-HSCT showed the same cytokine profiles compared to MBP-reactive T cells at baseline, with a greater capacity to secrete pro-inflammatory Th1 than Th2 cytokines (67).

#### Effector Memory

Mucosal-associated invariant T (MAIT) cells constitute a subset of unconventional T cells at the junction of innate and adaptive immune systems (68, 69). Human MAIT cells, 2 innate-like lymphocytes, express the semi-invariant TCR (TCR: iVa7.2-Ja33) and are selected by the Major histocompatibility complex (MHC)-related protein 1, MR1 on hematopoietic cells (70). In the adult, MAIT cells represent 10% of mature CD8^+^ or CD4^-^CD8^-^ (DN) T cells. This population plays an important role against bacterial, yeast, and viral infections (69, 71) recognizing antigens (Ags) released from microbial riboflavin (vitamin B2) synthesis (72). In human, MAIT cells were defined as CD161^hi^IL-18Ra^+^Vα7.2^+^γδ^-^CD3^+^ lymphocytes with effector memory phenotype CD45RA^-^CD45RO^+^CD62L^lo^CD122^dim^CD127^hi^CD95^hi^ (73), expression of the innate transcription factor promyelocytic leukemia zinc finger (PLZF), RAR-related orphan receptor gamma (RORγt), and intermediate levels of T-Box transcription factor TBX21 (T-bet) (74). This subset of lymphocytes expresses heterogeneous levels of NK receptors and chemokines (CCR6, CXCR6, CXCR4, and CCR9) that consent the migration to peripheral tissue especially intestine and liver (73). MAIT cells secrete high levels of granzyme B, TNFα, and IFNγ upon CD3 and CD28 stimulation and high levels of IL-17 after PMA–ionomycin stimulation (73). MR1-Ag-loaded tetramers have been used for the specific identification of subsets of MAITs (75). MAIT cells were detected in white matter and perivascular infiltrates of postmortem MS brain tissues and were radically depleted after non-myeloablative HSCT and remained nearly undetectable in the PB for the whole follow-up period of 2 years (66). MAIT cells are defined as the major producer of IL-17 in the CD8 compartment, and their ablation after HSCT implicated the attenuation of Th17 and Th1-Th17 cells (67). In addition, pro-inflammatory MAIT cells were depleted in patients with MS at 3, 6, and 12 months post-HSCT (38). Flow cytometry analysis of PB mononuclear cells (PBMC) isolated in pre- and post-HSCT and stimulated under Th17-polarizing conditions for 6 days demonstrated a lower frequency of Th17 (IFNγ^-^IL-17^+^) and Th1-17 (IFNγ^+^IL-17^+^) after immune reconstitution (67). Similar results were observed in CD8 T cell responses. IL-17-producing CD8 T (Tc17) responses (IFNγ^-^IL17^+^ CD8^+^ T cells) were lower than the baseline, whereas no difference was reported for type 1 CD8 T cell (Tc1) responses (IFNγ^+^IL17^-^ CD8^+^ T cells) (67). Accordingly, reduced levels of RoRγ-T (Th17 transcriptional factor) and an unchanged expression of T-bet (Th1 transcriptional factor) were detected in activated peripheral blood mononuclear cells (PBMC) in post-HSCT compared to the baseline (67). Those results were also confirmed by analysis of culture supernatants by ELISA. Lower levels of secretion of IL-17A were reported after-HSCT and associated with reduced levels of Th17-polarizing cytokines IL-1β and IL-6 and unchanged levels of TGFβ and IL-23 (67).

Decrement of Th1-17 T cells was confirmed in a further investigation (65). Examination of T helper cells by chemokine expression on CD4 memory T cells (65) showed that CXCR3^+^CCR6^-^Th1 effector memory cells increased at 6 months and began to decrease at 2 years without reaching the baseline values. Contrary to CXCR3^-^CCR6^+^Th17 effector memory cells that maintained the same levels during the follow-up, CXCR3^+^CCR6^+^ Th1-17 effector memory cells lowered to the baseline at month 6 and year 2. Polyfunctional (co-expressing TNFα and IL-2) IFNγ low- and high-producing CD8 T cells were examined over time. IFNγ low–producing cells increased at month 2 and returned to the baseline at years 1 and 2 compared to IFNγ high-producing CD8 T cells that remained the same at all the time points examined. Expression of programmed cell death protein-1 (PD-1) was observed to increase on CD4 and CD8 T cell populations at month 2 and to return to baseline levels at 1 and 2 years. The IFNγ low- and high-producing CD8 T cells showed a remarkable increased co-expression of activation markers CD57, CD38, and HLA-DR at month 2 which returned to baseline levels at years 1 and 2 (65).

#### Regulatory T Cells

Regulatory CD4 T cells including CD39^+^-expressing cells (CD4^+^CD25^hi^CD127^-^CD39^+^) (38) and Foxp3^+^ (CD4^+^CD25^hi^CD127^-^Foxp3^+^) (66) and CD8^+^CD57^+^ T cells increased after HSCT and non-myeloablative treatment (62, 65–67, 76). Memory CD4 T regs (CD25^+^CD127^low/-^CD45RA^-^) increased significantly at month 6 to return to baseline levels at 2 years post-HDIT/HSCT (65). CD8^+^ CD57^+^T cells constituted a great proportion of the CD8 T cell pool after HSCT with immunoregulatory function related to the ability to suppress CD4 T cell proliferation in cell coculture (66). Increases of cytotoxic T-lymphocyte antigen 4 (CTLA-4) on CD4 regulatory T cells and programmed cell death protein 1 (PD-1) on CD19 and CD8^+^CD57^+^cells were associated with positive outcomes post-HSCT. PD-1 signaling and regulatory T cells were described as biological mechanisms restoring immune tolerance post-HSCT in HALT-MS (76).

#### B Cells

Total B cells reduced modestly at 2 months and increased from the baseline level at 1 and 2 years post-HDIT/HSCT (65). Naive B cells constituted the predominant subset within the circulating B cells at 1 and 2 years post-transplantation. An immunophenotyping study by flow cytometry was conducted on PBMCs isolated from 28 patients enrolled for AHSCT with HDIT/HSCT before and after therapy in a follow-up of 72 months (76). CD19^+^ B cell counts increased at 18 and 24 months post-HSCT, mainly in patients responsive to HSCT (76). Most of them are naïve cells and expressed PD-1 (65, 76).

#### Oligoclonal IgG Bands

(OCBs) in the CSF are a biomarker of intrathecal B and plasma cell activation in patients with MS. In one study, CSF analysis of 4 patients pre and after HSCT revealed that OCBs reduced in the CSF at a rate consistent with reduced ongoing IgG synthesis rates (77). Other studies that reported OCBs remained largely unchanged (30, 34, 35, 78). Persistence but reduction of OCBs in the CSF was observed at 2 years of the 4-year patient follow-up with a reduction of CSF IgG levels (41). Long-term studies showed that OCB are decreased or disappeared. After HSCT, the IgG and IgM indices decreased and OCB were lower (79). A Swedish study with a 10-year follow-up after HSCT demonstrated that 60% of patients lost CSF OCB and only one patient (10%) had IgG above the normal levels and had relapse (80).

### Innate Immunity

#### NK Cells

The immune reconstitution of natural killer (NK) cells in the PB of patients with MS after receiving HSCT was investigated in 3 studies as reported (38, 66, 81). In all the studies, CD56 NK cells expanded by month 2. The frequencies of immunoregulatory CD56^hi^ NK cells (CD3^-^CD16^-^CD56^hi^) increased significantly by 3 months and remained high in a follow-up of 12 months post-HSCT (38). NK cells increased at 2 months and began returning to the baseline level by month 3 after HSCT (81). Frequencies of both the CD56^dim^ and CD56^bright^ NK cell subsets rose between month 3 and month 6 post-HSCT and remained elevated until month 18 and significantly higher at 12–18 months post-HSCT (81). The ratio NK bright cells (CD56^bright^/CD56 ^dim^) was 0.1 at the baseline, 0.6 from months 3 and 6, and dropped to baseline levels by 24 months. The rapid immune reconstitution of NK cells was associated with incomplete ablation or presence of NK cells in the graft. Patients with a greater increase in NK cells showed the greatest reductions in Th17 responses associated with NK-mediated immune suppression (81).

**Figure 1:**
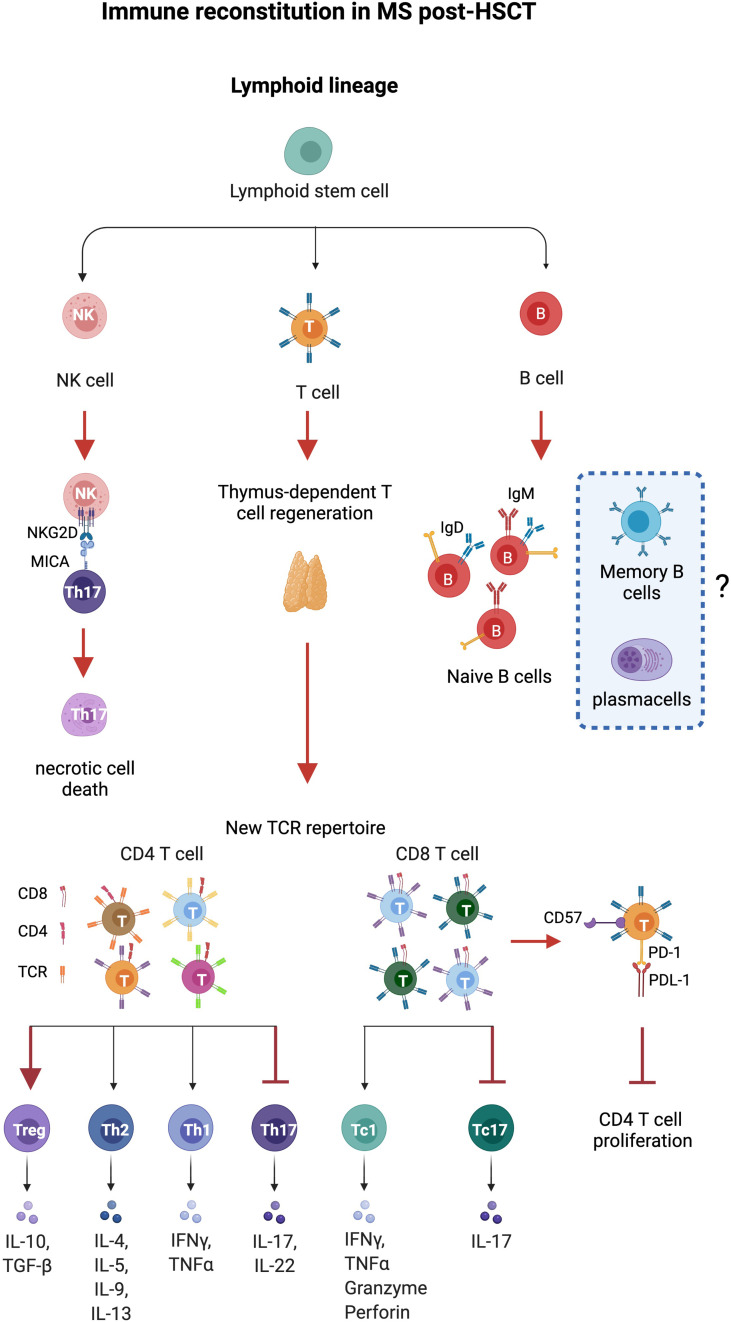
Immune reconstitution in MS after HSCT. Thymus-dependent T cell regeneration and immune regulation mediated by T and natural killer (NK) cells constitute the major identified pathways influencing immune reconstitution in patients with MS after HSCT. Thymus-derived CD4 T cells show a new, diverse repertoire and deletion of preexisting CD4 clones. CD8 T cells show an incomplete renewal of TCR repertoire suggesting expansion of residual or regenerated virus-specific clones. Increase of regulatory CD4^+^FOXP3^+^, CD8^+^CD57^+^T cells, and NK cells and eradication of pro-inflammatory Th17 and Th17-1 cells (MAIT) are observed after HSCT. NK cells induce necrotic cell death in Th17 and Th17-1 cells by the NKG2D pathway, while CD8^+^CD57^+^ cells suppress CD4^+^ T cell proliferation. Anergy is reported on CD8^+^ T cells that express high levels of CD57, a marker of senescence, and inhibitory effects are exerted by the immune checkpoint inhibitor PD-1. HSC, hematopoietic stem cells; CLP, common lymphocyte progenitor; NK regs, regulatory natural killer CD3-CD56^hi^. Figure created with BioRender.com.

Studies of immune reconstitution after myelo and non-myeloablative HSCT have reported 1) decrement of inflammatory subsets Th17 and Th17-1; 2) increment of regulatory populations represented by regulatory CD4 T cells, CD8^+^CD57^+^ T cells, and CD56^hi^NK cells; 3) renewal of the TCR repertoire in CD4 T cells associated with thymic output; and 4) resetting of the B cell compartment with an enrichment of naïve B cells. The kinetics of immune reconstitution after HSCT were reported as shown in **Table 2** while the relevant biological processes in the immune reconstitution in patients with MS post-HSCT are recapitulated as shown in **Figure 1**.

**Table 2:** Phenotype of human B, T, and natural killer (NK) cell subsets in the periphery.

Lymphocyte subpopulations	Phenotype	Perturbation in MS	Month 6 post-HSCT	Year 1 post-HSCT	Year 2 post-HSCT
**T cell subsets (CD3^+^)**					
CD4-naïve T cells	CD4^+^CCR7^+^CD45RA^+^		*^b^* ↓	≅	≅
CD4+ central memory (CD4^+^ T_CM_)	CD4^+^CCR7^+^CD45RA^-^	Detected in lesions, CSF	↓	≅	≅
Th1 central memory (Th1_CM_)	CD4^+^CCR7^+^CD45RA^-^CCR6^-^CXCR3^+^	Detected in lesions, CSF	≅	≅	≅
Th17 central memory (Th17_CM_)	CD4^+^CCR7^+^CD45RA^-^CCR6^+^CXCR3^-^	Detected in lesions, CSF	↓↓	↓↓	≅
Th1Th17 central memory (Th1Th17_CM_)	CD4^+^CCR7^+^CD45RA^-^CCR6^+^CXCR3^+^	Detected in lesions, CSF	↓↓	↓↓	≅
CD4+ effector memory T cell (CD4^+^ T_EM_)	CD4^+^CCR7^-^CD45RA^-^	Detected in lesions, CSF	↑↑	≅	≅
Th1 effector memory (Th1_EM_)	CD4^+^CCR7^-^CD45RA^-^CCR6^-^CXCR3^+^	Detected in lesions, CSF	≅	≅	≅
Th17 effector memory (Th17_EM_)	CD4^+^CCR7^-^CD45RA^-^CCR6^+^CXCR3^-^	Detected in lesions, CSF	↓↓	↓↓	↓↓
Th1Th17 effector memory (Th1Th17_EM_)	CD4^+^CCR7^-^CD45RA^-^CCR6^+^CXCR3^+^	Detected in lesions, CSF	↓↓	↓↓	↓↓
Terminal differentiated effector memory CD4+ T cell (T_EMRA_)	CD4^+^CCR7^-^CD45RA^+^	Detected in lesions, CSF	*^c^* ↑	*^d^* ≅	≅
Regulatory CD4^+^T cells	CD4^+^CD25^hi^CD127-FOXP3^+^/ CD4^+^CD25^hi^CD127^-^CD39^+^	Detected in lesions, CSF	↑↑↑	↑↑	≅
CD8^+-^naïve T cell	CD8^+^CCR7^+^CD45RA^+^		↓	≅	≅
CD8^+^ central memory T cell (CD8^+^ T_CM_)	CD8^+^CCR7^+^CD45RA^-^	Detected in lesions, CSF	↓	≅	≅
CD8^+^ effector memory T cell (CD8^+^ T_EM_)	CD8^+^CCR7^-^CD45RA^-^	Detected in lesions, CSF	↑↑	≅	≅
Cytolytic CD8+ effector T cells (Tc1) secrete IFN-γ		Detected in lesions, CSF	≅	≅	≅
Cytolytic CD8^+^ effector T cells (Tc17) secrete IL-17		Detected in lesions, CSF	↓	↓	↓
Cytolytic CD8^+^ T cells (Tc17-1) secrete IFNγ and IL-17		Detected in lesions, CSF	↓	↓	↓
MAIT cells	CD8^+^CD161^hi^TCRVa7.2^+^IL-18R^+^CD45RA^-^CD127^hi^CD95^hi^	Detected in lesions, CSF	↓↓↓↓	↓↓↓↓	↓↓↓↓
Terminal-differentiated effector memory CD8^+^ T cell (T_EMRA_)	CD8^+^CCR7^-^CD45RA^+^	Detected in lesions, CSF	↑	≅	≅
**B-cell subsets**					
Transitional B cells	IgD^lo/-^IgM^+^CD10^hi^CD24^hi^CD38^hi^	Dysfunctional	*^e^*?	?	?
Naïve B cell subsets	IgD+CD27+		↓	↑↑	↑↑
	CD45RB^-^CD27^-^CD38^-^CD305^+^IgD^+^CD73^-^				
	CD45RB^-^CD27^-^CD38^-^IgM^+^CD73^+^				
Naïve B10 cell subsets			?	?	?
	CD19^+^CD24^hi^CD27^+^	Abnormal			
	CD19^+^CD27^+^	↓↓			
	CD19^+^CD38^hi^				
Switched memory B cells (Bmems)	IgD^-^CD27^+^		↓	↓	↓
	CD19^+^CD20^hi^CD45RB^-^CD27^-^CD95^-^CD21^-^CD38^-^CD73^-^CD4^lo^IgG^hi^CD185^-^CD184^-^PD-1^+^CD11c^+^T-bet^+^	↑↑			
	CD45RB^+^CD27^+^CD73^+^IgG^+^/IgA^+^	-Antigen presenting cells capacity (self-antigens)? promoting autoreactive cell responses, -Increased release of GM-CSF,IL-6,TNFα and lymphotoxin-α upon BCR and CD40 engagement, -Bmems present RASPGRP2-derived epitopes that are targeted on Neuron by CD4 T cells, -Increased of Bmems in the CNS that contribute to generation of ectopic lymphoid structures (ELS).			
	CD45RB^+^CD27^+^CD73^-^IgA^+^CD9^+^CD22^-^				
Long-lived Bmems	CD45RB^-^CD27^+/-^IgG^+^/IgA^+^ CD73^+/-^CD183^+/-^				
Effector Bmems	CD95^+^IgG^+^/IgA^+^				
Antibacterial specificity	CD27^-^IgA^+^				
Aged or exhausted Bmems	CD27^-^IgG3^+^/IgG2^+^				
Late memory B cells	IgD^-^CD27^-^		↑	↓	↓
Plasma cell subsets		-Contribute to large amount of high-affinity antibodies, high levels of immunoglobulin IgG and IgM in the cerebrospinal liquid,	≅	≅	≅
Short-lived plasma cells	CD19^+^CD38^+^CD27^hi^IgG/IgA^+^IgM^+^				
Long-lived plasma cells	CD19^+^CD38^+^CD27^hi^CD184^+^IgG/IgA^+^IgM^+^				
Regulatory plasma cells	CD19^+^CD138^+^				
**NK subsets**					
Regulatory NK cells	CD3^-^CD56^hi^	Dysregulated or impaired	↑↑	↑↑	≅
Cytotoxic NK cells	CD3^-^CD56^dim^	Dysregulated or impaired	↑↑	↑↑	≅

^c^Aarrow ↑= increase, ^b^arrow ↓= decrease, ^d^≈ = approximaly equal, ^e^?= unknown.

Subsets of lymphocytes that are described as “pathogenic” in MS and kinetics of immune reconstitution post-HSCT compared to the baseline (pre-treatment) in MS.

### New Questions and Perspective on Future Studies

#### Stem Memory Cells

**Figure 2:**
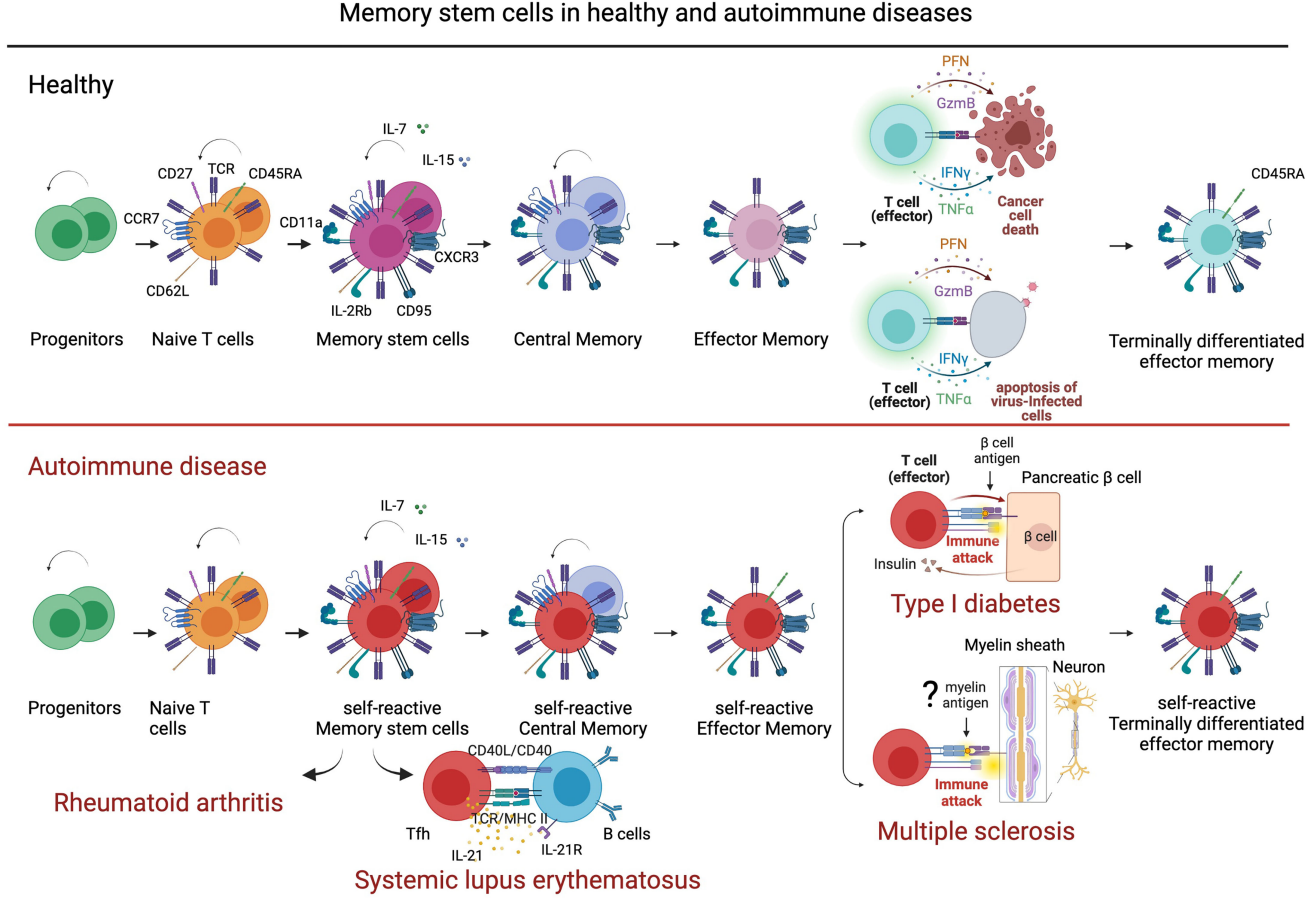
Stem memory cells in healthy and autoimmune disease. Stem memory cells constitute a subset of T cells with self-renewal and multipotent capacity. Generated from naïve cells, stem memory cells develop into memory subsets including central memory and effector memory. Stem memory cells express a naïve-like phenotype (CD45RA^+^, CCR7^+^, CD62L^+^, CD27^+^, CD28^+^, and IL7Ra^+^) and memory markers (CD95^+^, CXCR3^+^, IL-2Rb^+^, CD58^+^, and CD11a^+^), which vary during further differentiation. While essential to immunological memory in healthy immune system, memory stem cells also represent a reservoir of autoreactive clones in autoimmune disease. For example, in type I diabetes (TDI) self-reactive β-cell-specific CD8 T cells maintain a memory stem cell phenotype that favors a persistent production of pathogenic clones. In systemic lupus erythematosus, memory stem cells differentiate easily into T follicular helper cells (Tfh) that contribute to B cell differentiation and antibody production. In multiple sclerosis, stem memory cells recognizing, hypothetically, myelin antigens could represent a supply of pro-inflammatory and cytotoxic cells targeting myelin and damaging neurons. Figure created with BioRender.com.

Self-reactive memory B and T cells have a crucial role in the pathogenesis of MS as reported in clinical–pathological and functional studies (82–86). The knowledge of molecular mechanisms that regulate or produce immunological memory could be essential for the development of immune therapeutic intervention in MS. Long-lived memory cells are generated from adaptive immunity and guarantee a long-lasting protection against microbes and tumors. However, immunological memory can favor chronic inflammation and contribute to the development and maintenance of immune-mediated diseases. Among memory T cell subsets, stem memory T cells (Tscm) have the capacity of self-renewal and multipotency. Tscm differentiate *in vitro* from naïve T cells by triggering Wnt-b-catenin signaling during T cell priming (87, 88). Tscm constitute a small proportion of circulating CD4 and CD8 T cells (>2%–3%) with naïve-like phenotypes (CD45RA^+^, CCR7^+^, CD62L^+^, CD27^+^, CD28^+^, and IL-7Rα^+^) and memory markers (CD95^+^, CXCR3^+^, IL-2Rβ^+^, CD58^+^, and CD11a^+^). Tscm showed either memory and naïve T cell properties, including a low level of TCR rearrangement excision circles (TREC), ability to acquire rapidly effector function in response to antigens and differentiation in memory subtypes, dependence on IL-15 and IL-7 for homeostatic turnover, asymmetric proliferation typical of multipotent cells, homing to lymph node and tumor, and viral antigen specificities (89). Compared to naïve T cells, Tscm are maintained by extensive proliferation and display a higher level of telomerase activity that gives them the HSC features (90). Moreover, Tscm lymphocytes can differentiate directly from naive precursors infused within the graft (91), and the extent of Tscm generation correlates with IL-7 serum levels. In agreement with recent findings, Tscm could constitute the precursors and the reservoir of autoreactive clones causing autoimmune disorders. Th17 cells, described as pathogenic in chronic autoimmunity (92), have been highlighted to persist longer and expand more efficiently than Th1-derived cells *in vivo* endowed of the molecular program of survival and self-renewal. Long-term CD8 T cell responses to yellow virus are detectable over 25 years after vaccination and show a naïve-like phenotype CD45RA^+^ CCR7^+^ with a characteristic of memory cells (93, 94). Therefore, the relevance of Tscm in the differentiation of long-term memory T cells was obtained monitoring over several years T lymphocytes, genetically modified to express thymidine kinase (TK) suicide gene (95, 96). Tscm have been investigated in immune-mediated disorders. In type I diabetes (TID), beta-cell-specific T cells persist for long periods generating an antigenic response to pancreatic islet transplant recipients. The mechanisms that preserve effector and memory features in beta-cell-specific CD8 T cells through the life of type I diabetic patients have been investigated (97). Single cell ATAQ-seq showed the coexistence of naïve and effector–associated epigenetic programs in tetramer positive beta-cell-specific CD8 T cells isolated from patients with type I diabetes (97). This hybrid condition supports the hypothesis that a long-lived population of cells that retain effector potential may preserve a sustained self-reactive state. Moreover, beta cell-specific CD8 T cells isolated and stimulated for 14 days maintain multipotency-associated epigenetic programming after undergoing extensive antigen-dependent proliferation (97). CD161^hi^ CD8^+^ Tscm cells have antiviral specificities and regenerate the antiviral memory pool after chemotherapy displaying futures of memory stem cells (98, 99). CD4^+^ Tscm cells in systemic lupus erythematosus (SLE) patients showed a gene profile facilitating T follicular helper (Tfh) cell differentiation and antibody production (100). Tfh has been described dysfunctional in SLE and to help B cells in the generation of germinal centers and production of high-affinity and isotype-switched antibodies (101). Moreover, levels of CD4 Tscm increase and correlate with disease activity in patients with rheumatoid arthritis (RA). Receiving a pro-survival signaling from TNFR2, CD4 Tscm cells undergo oligoclonal TCR repertoire expansion that constitutes a reservoir of autoreactive cells (102). The ability of Tscm to differentiate in Tfh and participate to high-affinity and isotype-switched antibodies confers to this T cell subset a potential pathogenic role in MS. Differentiation and self-renewal of memory stem cells in the healthy immune system and in autoimmune diseases are illustrated in **Figure 2**. The contribution of B cells to the pathogenesis of MS has been recently reviewed in depth and has highlighted the need for further investigation of B immune reconstitution (85).

### Secondary Autoimmunity

Development of a new (secondary) autoimmune disease in patients with a preexisting (primary) autoimmune condition has been reported after treatment with autologous HSCT at rates ranging from 2% to 14% (103). The rates of secondary autoimmune diseases, termed henceforth 2ndADs, following treatment with ALEM are higher, requiring special monitoring in patients with MS and cautioning against the use of ALEM as lymphodepleting treatment in HSCT regimes. The occurrence, risk factors, and immunological mechanisms of 2ndADs after treatment with HSCT have been the specific focus of a recent publication in which the authors suggest that an imbalance of effector and regulatory T and B cells, or delayed reconstitution of the latter, may underlie the occurrence of the adverse event (103). This notion further supports the rationale of studies of immune reconstitution after HSCT, particularly including the kinetics of reconstitution of regulatory and effector cells and examining the correlation with any 2ndADs.

### Vaccination Following HSCT

HSCT recipients often lose immunity to vaccine-preventable diseases following transplantation and are therefore considered “never vaccinated” (104, 105). In patients who have received an autologous HSCT, pathogen-specific immune reconstitution in the absence of vaccination can be poor even several years post-transplant, with 98.2%, 100%, and 34.5% showing titers below protective thresholds for against diphtheria, *Streptococcus pneumoniae*, and measles virus, respectively (106). International and EBMT guidelines therefore recommend commencement of a routine vaccination program at 3–6 months after HSCT (6, 107). Vaccine responses can, however, be suboptimal in the first year post-HSCT, with minimal immunogenicity to influenza vaccines seen during the first 6 months (108). Similarly, emerging data suggest that responses to SARS-CoV-2 mRNA vaccines are lower during the first 12 months post-HSCT (109). However, preliminary reports in HSCT recipients suggest around 60% positivity (110) rate for SARS-CoV-2 antibodies post-vaccination, and the presence of a memory T cell response (111) elicited by a second dose of vaccine.

Post-HSCT vaccination offers opportunity to explore whether beneficial pathogen-specific immunity can be induced while maintaining the broad post-transplant immune tolerance associated with favorable outcomes in MS. It is possible that the degree of tolerance present may be associated with the magnitude of response to specific vaccines, especially during the first few months after transplant. Nevertheless, post-HSCT vaccine responses may be superior to those seen in patients who have received certain DMTs who have an ongoing immunosuppressive state. Although there is currently limited evidence for relapse of autoimmune conditions post-HSCT due to vaccination-induced disruption of immune tolerance, it is important to establish the safety of vaccine programs in patients with MS who have undergone HSCT, particularly with more novel vaccine technologies.

### Vaccination and DMT

Immune response to vaccinations could be hampered by immune-suppressive treatments. Vaccine humoral immune responses were reduced in patients treated with NAT and significantly impaired by anti-CD20 monoclonal antibody therapies. The timing of vaccination played an important role in those treated with ALEM (112). The vaccine-induced long-term immunologic memory against pathogens relies both on humoral and on cellular immune responses. Despite patients treated with anti-CD20 antibodies showing significantly reduced humoral response to SARS-CoV-2 mRNA vaccination, as compared to untreated patients (113), they generated robust CD4 and CD8 T cell responses suggesting that vaccinating B cell-deficient patients are still likely to provide some measure of immunity to SARS-CoV-2 (114).

## Conclusions

Treatment with autologous HSCT for patients with aggressive RRMS has been demonstrated to be highly effective (37, 115) with a significant reduction of treatment related mortality over time (currently < 1%). The American Society of Bone Marrow Transplantation (ASBMT) and the European Bone Marrow Transplantation (EBMT) Autoimmune Diseases Working Party (ADWP) have endorsed HSCT as a “standard of care” treatment for DMT-resistant poor-prognosis inflammatory MS (7, 116). Infection risk related to chronic or cyclic immune suppression from DMTs is initially low but accumulates over time with long term, while in HSCT the risk is early after transplantation with patients benefiting from a lack of long-lasting immune suppression.

Results from studies of immune reconstitution post-HSCT showed a change in immune profile suggesting a shift toward tolerance, characterized by depletion of pro-inflammatory TH1/17 cells, senescence of terminally differentiated effector memory cells, increase of naïve cells with new TCR repertoire, and regulatory profile in early stage. Despite the knowledge of the T cell compartment, little information is available for B cell immune reconstitution.

Tscm cells represent an early stage of memory cells with propriety of self-renewal and effector cells. Their susceptibility to generate chronic inflammation and autoimmune disease has spotlighted the necessity to investigate this population in MS. Studies focused on defining the differentiation pathway, transcriptome profiles, and characterization of molecules associated with stem and effector-like function before and after HSCT could help to understand the mechanisms of action of the procedure and potentially elucidate the reason for the less common occurrences of disease persistence or reactivation, and 2ndADs.

## Author Contributions

MC and PM conceptualized the manuscript. MC wrote and revised the manuscript. AG and GB wrote the paragraphs: “Disease modifying treatments in MS”, “HSCT versus DMT”, and “Vaccination and DMT” and revised the manuscript. TdS wrote the paragraph “Vaccination following HSCTA” and revised the manuscript. TA, RG, JS, and BS critically reviewed the manuscript for important intellectual content and edited the manuscript. PM supervised, reviewed, and revised the manuscript. All authors contributed to the article and approved the submitted version.

## Funding

This review was funded by the European Society for Blood and Marrow Transplantation Leiden Netherland Reference: ADWP.

## Conflict of Interest

PM reports no conflict of interest. He discloses travel support and speaker honoraria from unrestricted educational activities organized by Novartis, Bayer HealthCare, Bayer Pharma, Biogen Idec, Merck Serono, and Sanofi Aventis. He also discloses consulting to Magenta Therapeutics and Jasper Therapeutics. JS declares honoraria for an advisory board from MEDAC, and as an IDMC member for a trial supported by Kiadis Pharma, all outside the submitted work. RG discloses honoraria for speaking from educational events supported by Biotest, Pfizer, and Magenta.

The remaining authors declare that the research was conducted in the absence of any commercial or financial relationships that could be construed as a potential conflict of interest.

## Publisher’s Note

All claims expressed in this article are solely those of the authors and do not necessarily represent those of their affiliated organizations, or those of the publisher, the editors and the reviewers. Any product that may be evaluated in this article, or claim that may be made by its manufacturer, is not guaranteed or endorsed by the publisher.
